# Supplementary material for: Visible to mid-infrared giant in-plane optical anisotropy in ternary van der Waals crystals
Source: Nat Commun. 2023 Oct 24;14:6739. doi: 10.1038/s41467-023-42567-x (PMC10598000; doi:10.1038/s41467-023-42567-x)
Supplement: Supplementary file 1 — Supplementary Information [file 41467_2023_42567_MOESM1_ESM.pdf]

Supplementary Information

Visible to Mid-infrared Giant In-Plane Optical Anisotropy in Ternary Van der Waals Crystals

Yanze Feng,[1,2,//] Runkun Chen,[3,4,//] Junbo He,[5, //] Liujian Qi,[1,2] Yanan Zhang,[1,2] Tian Sun,[3] Xudan Zhu,[5] Weiming Liu,[5] Weiliang Ma,[3] Wanfu Shen,[6] Chunguang Hu,[6] Xiaojuan Sun,[1,2] Dabing Li,[1,2,*] Rongjun Zhang,[5,*] Peining Li,[3,*] Shaojuan Li[1,2,*]

[1]State Key Laboratory of Luminescence and Applications, Changchun Institute of Optics, Fine Mechanics and Physics, Chinese Academy of Sciences, Changchun, Jilin 130033, P. R. China

[2]University of Chinese Academy of Sciences (UCAS), Beijing 100049, P. R. China

[3]Wuhan National Laboratory for Optoelectronics & School of Optical and Electronic Information, Huazhong University of Science and Technology, Wuhan 430074, China

[4]State Key Laboratory of Structural Chemistry, Fujian Institute of Research on the Structure of Matter, Chinese Academy of Sciences, Fuzhou, 350002, China

[5]Department of Optical Science and Engineering, Shanghai Frontiers Science Research Base of Intelligent Optoelectronics and Proception, Institute of Optoelectronics, Fudan University, Shanghai 200433, China

[6]State Key Laboratory of Precision Measuring Technology and Instruments, Tianjin University, Weijin Road 92, Nankai District, Tianjin 300072, China

*Corresponding author. E-mail: (D. L.) lidb@ciomp.ac.cn; (R. Z.) rjzhang@fudan.edu.cn; (P. L.) lipn@hust.edu.cn and (S. L.) lishaojuan@ciomp.ac.cn;

[//] These authors contributed equally.

Supplementary Table 1. Comparison of the in-plane structural anisotropy in different categories of van der Waals (vdW) materials.

Materials	a（Å）	b（Å）	c（Å）	In-plane structural anisotropy	Ref.
Ta_2NiS_5	3.415	12.146	15.097	c/a=4.42	[1]
Ta_2NiSe_5	3.496	12.829	15.641	c/a=4.474	[1]
$TaIrTe_4$	3.77	12.421	13.184	b/a=3.295	[2]
1T’-$MoTe_2$	6.33	3.47	13.86	a/b=1.82	[3]
$PdSe_2$	5.7457	5.8679	7.6976	b/a=1.021	[4]
PdS_2	5.46	5.541	7.531	b/a=1.015	[4]
MoO_3	13.865	3.698	3.963	a/c=3.499	[5]
WTe_2	3.496	6.282	14.07	b/a=1.8	[6]
quasi 1D-TiS_3	4.9728	3.4055	8.8146	a/b=1.46	[7]
quasi 1D-ZrS_3	5.1107	3.6179	8.9725	a/b=1.413	[7]
ReS_2	6.45	6.39	6.40	a/b=1.009	[8]
$ReSe_2$	6.71	6.62	6.74	a/b=1.014	[9]
GeS	4.29	3.64	10.42	a/b=1.18	[10]

Supplementary Note 1: Anisotropic structural characterization of Ta_2NiS_5 crystals.

We first determined the in-plane crystal axis (*a*-axis and *c*-axis) of Ta_2NiS_5, which is beneficial to the further study of anisotropic optical properties of Ta_2NiS_5. **Supplementary Figure 1a** shows the optical microscope images of the Ta_2NiS_5 flakes. We used angle-resolved polarization Raman spectroscopy to determine the crystal orientation of Ta_2NiS_5 sample. As shown in **Supplementary Figure 1a**, the long-axis of the sample is defined as the y direction, and the x direction is perpendicular to the y-axis. The angle θ represents the incident angle between the incident light polarization directions and sample y-axis, which is varied from 0 to 360°. **Supplementary Figure 1c** displays energy dispersive spectroscopy (EDS) spectra of the Ta_2NiS_5 flake. We can see that the element ratios of Ta/Ni/S (2:1:5) are approximate to their molar ratio. **Supplementary Figure 1d** shows the characteristic Raman peaks of Ta_2NiS_5. The Raman active modes of 39.9, 63.1, 123.5 and 143.5 cm^{-1} are assigned to ${}^1A_g$, B_{2g}, ${}^2A_g$, and ${}^3A_g$ respectively, which is consistent with previous reports[11,12]. Under the parallel-polarization configuration, the intensity of different Raman modes varies periodically with changing rotation angle, indicating strong in-plane vibration anisotropy (**Supplementary Figure 1e**). It can be clearly seen from **Supplementary Figure 1f** that the ${}^3A_g$ mode is minimized when the laser polarization direction is parallel to the y direction. According to the previous literature report[11], we can conclude that the y direction corresponding to the *a*-axis of the crystal. From the above analysis, a quick way to determine the orientation of the crystal is to observe its shape, which is usually elongated along the *a*-axis.

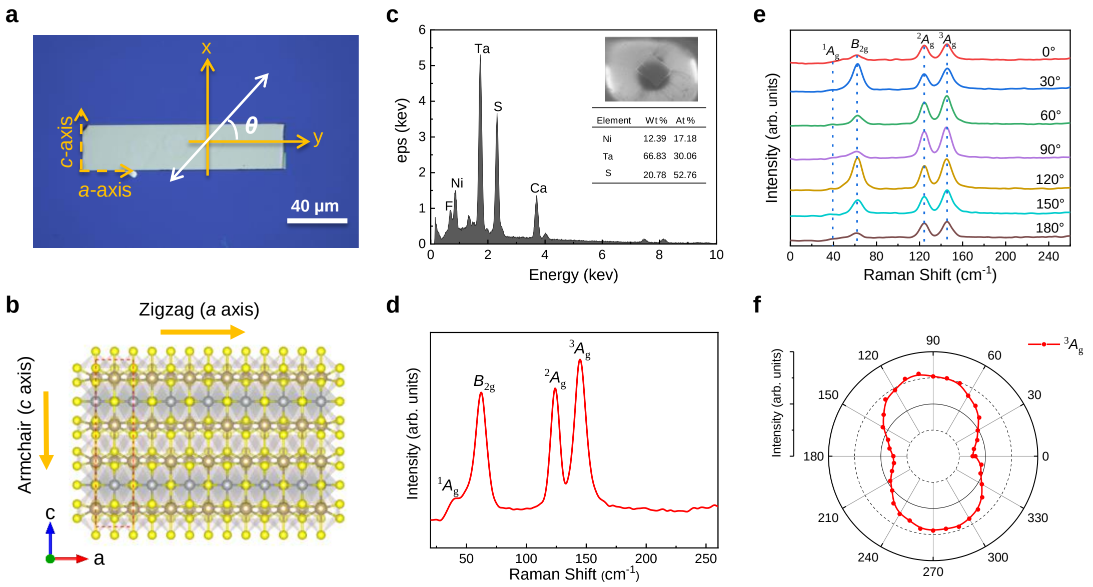

Supplementary Figure 1. Characterizations of the Ta_2NiS_5 crystals. a Optical microscope images of the exfoliated Ta_2NiS_5 flakes. **b** Crystal structures of Ta_2NiS_5. The red dash region represents the unit cell of Ta_2NiS_5. **c** Energy dispersive spectroscopy (EDS) results of an exfoliated Ta_2NiS_5 flake on CaF_2 substrate. Insets show morphology and the component of the flake. **d** Raman spectra of Ta_2NiS_5 flakes. **e** The angle-resolved polarized Raman spectra of Ta_2NiS_5. **f** The peak intensity (${}^3A_g$ mode) as a function of polarization angle.

Supplementary Note 2: Spectroscopic ellipsometry measurement and analysis

The spectroscopic ellipsometry measurements were taken under room temperature at an incident angle of 65°. And the spot size of the ellipsometer is 60 μm×25 μm, which guarantees the valid spectroscopic ellipsometry signal from our samples. To gain physical information of the in-plane anisotropy of the Ta_2NiS_5 flakes, we rotated the sample's azimuthal orientation by every 10 degrees, from 0 to 360 degrees, to measure the ellipsometric data, as shown in **Supplementary Figure 2**.

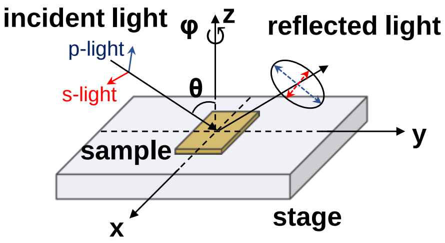

Supplementary Figure 2. Schematic diagram of the spectroscopic ellipsometry measurements with varying in-plane azimuth angles. θ is the angle of incidence. φ is the in-plane azimuth angle, which is defined as the angle between the incident plane and the horizontal plane of the sample. The sample is illuminated by a collimated and polarized beam containing *p*- and *s*-components.

By measuring the changes in the polarization state of light after reflection from the sample surface, the optical properties can be analyzed by spectroscopic ellipsometry. The changes are represented by the ellipsometric parameters Psi (Ψ) and Delta (Δ), which are related to ratio of the *p*- and *s*-polarization components as follows[13]:

$$\rho = r_p / r_s = \tan(\Psi)\exp(\mathrm{j}\Delta) \tag{1}$$

where r_p and r_s denote the Fresnel reflection coefficients of *p*- and *s*-polarized light, respectively.

For the spectroscopic ellipsometry analysis, a four-phase model consisting of the $Si/SiO_2/Ta_2NiS_5$/air ambient was established. The azimuthal angle-dependent spectroscopic ellipsometry spectra is shown in **Supplementary Figure 3.** Considering

the uniformity of the surface of our sample observed under optical microscopy, the roughness layer was not introduced during the analysis.

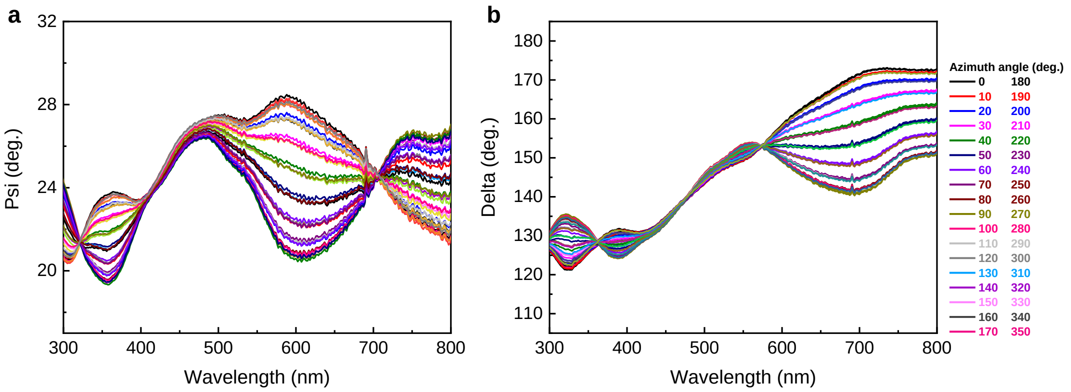

Supplementary Figure 3. **Measured ellipsometry spectra with different azimuthal angle.** The azimuthal angle-dependent Psi (a) and Delta (b) spectra with azimuthal angle from 0-350 degree by step of 10 degrees.

To unambiguously extract the dielectric functions of the Ta_2NiS_5 layer, we first employed the point-by-point method[14], which is a mathematical inversion process that requires the pre-knowledge of the thickness of each layer. The ellipsometric equation of the four-phase structural model can be expressed as:

$$\tan[\Psi(\lambda)]\exp[j\Delta(\lambda)] = f(\varepsilon_{Si}, \varepsilon_{SiO_2}, \varepsilon_{Ta_2NiS_5}, \varepsilon_{Air}, d_{SiO_2}, d_{Ta_2NiS_5}, \theta, \lambda) \tag{2}$$

The dielectric function of Si (ε_{Si}), SiO_2 (ε_{SiO_2}) are adopted from previous literature[15]. And the thickness of thermal SiO_2 layer was pre tested by spectroscopic ellipsometry on the clean substrate and its value is 290 nm which is fixed in the following spectroscopic ellipsometry analysis. The thickness of the Ta_2NiS_5 nanosheet was determined by the atomic force microscope (AFM) measurement, which is consistent with the results (421.0±0.5 nm) of later Lorentz model fitting. Therefore, with two measured parameters ψ and Δ at each wavelength, the ε_1 and ε_2 of Ta_2NiS_5 can be directly calculated through point-by-point method.

For the validation of the Kramers-Kronig consistency of the mathematical inversion result, we also considered the Lorentz model:

$$\varepsilon = \varepsilon_1 + j\varepsilon_2 = 1 + \sum_{i=1} \frac{A_i E_i}{E_i^2 - E^2 - j\Gamma_i E} \tag{3}$$

where E is the photon energy, and A_i, E_i, and Γ_i are the amplitude, center energy and damping coefficient of each oscillator.

The extracted dielectric functions of the point-by-point method and Lorentz model are displayed in **Supplementary Figure 4**. The fitting results of the two methods at different azimuth angles show very good agreement. The above results indicate that the spectroscopic ellipsometry is a precise and non-destructive method to identity the optical features of anisotropic vdW materials.

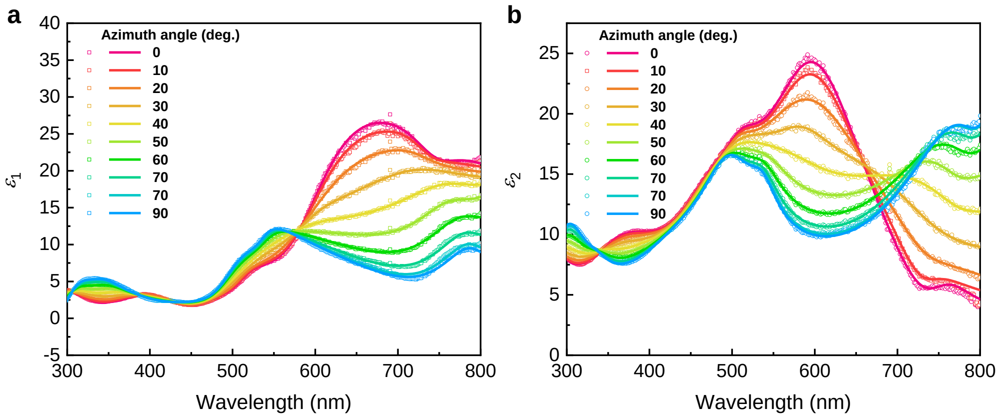

Supplementary Figure 4. Dielectric functions of Ta_2NiS_5 in visible range. The real **a** and imaginary **b** part of dielectric functions extracted by the point-by-point method (unfilled symbols) and Lorentz model (solid lines).

Identify of the crystallographic features from spectroscopic ellipsometry data

For clarity of the crystallographic features from spectroscopic ellipsometry spectra, **Supplementary Figure 5a and 5b** displays the azimuthal angle-dependent Psi and Delta spectra with azimuthal angle from 0 degree (*c*-axis) to 90 (*a*-axis). Significant differences in the spectroscopic ellipsometry data between the two crystallographic orientations can be observed. Thereby, spectroscopic ellipsometry offers an unambiguous and non-destructive method to identify the crystallographic features of the Ta_2NiS_5.

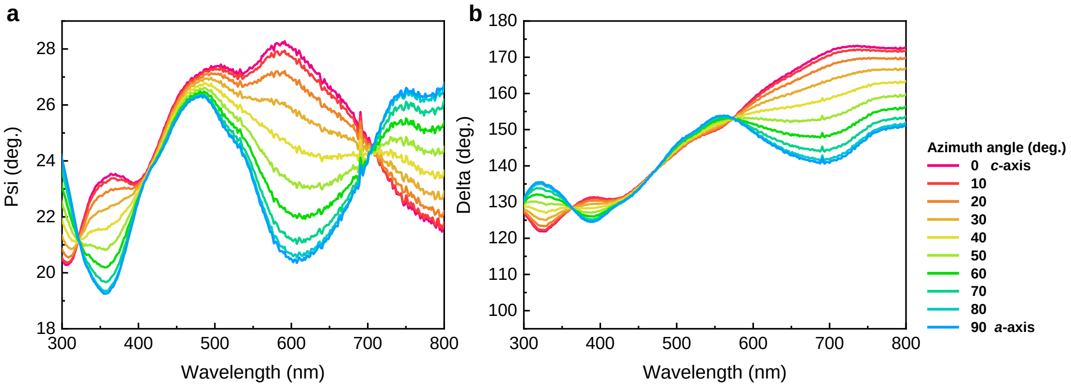

Supplementary Figure 5. Identify of the crystallographic features from spectroscopic ellipsometry. The azimuthal angle-dependent **a** Psi and **b** Delta spectra with azimuthal angle in the range of 0-90 degree.

Supplementary Note 3: Extracting Mid-infrared (MIR) optical constant of Ta_2NiS_5 from Fourier transform infrared spectroscopy (FTIR) spectra

The complex in-plane dielectric of Ta_2NiS_5 in MIR range was extracted by fitting the experimental reflection spectrum with the calculated ones. The reflection of a flake sample with an unpolarized incident light can be calculated by

$$R = \frac{(r_p)^2 + (r_s)^2}{2} \tag{4}$$

$$r_p = \frac{r_p^{12} + t_p r_p^{23}}{1 + t_p r_p^{12} r_p^{23}} \tag{5}$$

$$r_s = \frac{r_s^{12} + t_s r_s^{23}}{1 + t_s r_s^{12} r_s^{23}} \tag{6}$$

where r_p and r_s are the Fresnel's reflectivity coefficients of the flake sample for *p*- and *s*-polarized light respectively, $r_{p,s}^{12}$ and $r_{p,s}^{23}$ are the Fresnel's reflectivity coefficients of the first interface between air and sample flake and the second interface between sample flake and substrate for *p*- and *s*- polarized light respectively, $t_{p,s}$ is the Fresnel's transmissivity coefficient of the first interface for *p*/*s*-polarized light. For more details,

$$r_p^{12} = \frac{\sqrt{1 - \sin^2\theta / \varepsilon_b} - \sqrt{\varepsilon_a}\cos\theta}{\sqrt{1 - \sin^2\theta / \varepsilon_b} + \sqrt{\varepsilon_a}\cos\theta} \tag{7}$$

$$r_p^{23} = \frac{\sqrt{\varepsilon_a}\sqrt{1 - \sin^2\theta / \varepsilon_s} - \sqrt{\varepsilon_s}\sqrt{1 - \sin^2\theta / \varepsilon_b}}{\sqrt{\varepsilon_a}\sqrt{1 - \sin^2\theta / \varepsilon_s} + \sqrt{\varepsilon_s}\sqrt{1 - \sin^2\theta / \varepsilon_b}} \tag{8}$$

$$r_s^{12} = \frac{\cos\theta - \sqrt{\varepsilon_c - \sin^2\theta}}{\cos\theta + \sqrt{\varepsilon_c - \sin^2\theta}} \tag{9}$$

$$r_s^{23} = \frac{\sqrt{\varepsilon_c - \sin^2\theta} - \sqrt{\varepsilon_s - \sin^2\theta}}{\sqrt{\varepsilon_c - \sin^2\theta} + \sqrt{\varepsilon_s - \sin^2\theta}} \tag{10}$$

$$t_p = e^{4\pi i\omega d\sqrt{\varepsilon_a}\sqrt{1 - \sin^2\theta/\varepsilon_b}},\ t_s = e^{4\pi i\omega d\sqrt{\varepsilon_c - \sin^2\theta}} \tag{11}$$

where θ is the angle of incident light, $\varepsilon_{a,b,c}$ is the dielectric tensor of Ta_2NiS_5

sample, ε_s is the dielectric constant of substrate, d is the thickness of sample flake.

To fitting the experiment reflection spectrum, we used a genetic algorithm (GA) for the evaluation of parameters of **Supplementary Equation (4)**. As a result, the in-plane dielectric model parameters of Ta_2NiS_5 are shown below:

Supplementary Table 2. In-plane dielectric model parameters of Ta_2NiS_5.

	ε_∞	ω_p (cm^{-1})	γ_D (cm^{-1})	f_1 (cm^{-1})	ω_1 (cm^{-1})	γ_1 (cm^{-1})	f_2 (cm^{-1})	ω_2 (cm^{-1})	γ_2 (cm^{-1})
ε_c	10.74	1155.75	457.82	15223.34	7036.87	177.20	2839.66	3738.96	1584.04
ε_a	12.35	1545.28	461.85	14228.04	5137.17	788.83	1735.26	3047.70	1121.15

Then, the anisotropic in-plane permittivity of Ta_2NiS_5 can be described with the Drude-Lorentz (DL) model

$$\varepsilon_{a,c}(\omega) = \varepsilon_\infty - \frac{\omega_p^2}{\omega^2 - i\gamma_D\omega} + \sum_i \frac{f_i^2}{\omega_i^2 - \omega^2 + i\gamma_i\omega} \tag{12}$$

where ε_∞ is the high frequency dielectric constant, the second term represents the free carriers contribution, ω_p and γ_D are the plasma frequency and free carriers damping rate respectively, and the last term represents the Lorentzian oscillator contribution, f_i, ω_i and γ_i are the strength, oscillation frequency and damping rate of the i-th Lorentzian oscillator respectively.

Supplementary Figure 6 shows the experimental FTIR reflection spectrum of another Ta_2NiS_5 flake and the calculated reflection spectrum using above model parameters. The good agreement further verified the applicability the model parameters we have used.

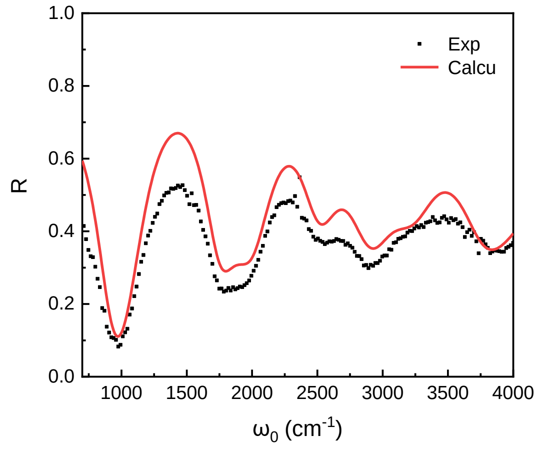

Supplementary Figure 6. Far-field Fourier transform infrared spectroscopy (FTIR) and calculated reflectance spectra of 1.15-μm-thick Ta_2NiS_5 on CaF_2 substrate.

Supplementary Note 4: Giant dichroism of Ta_2NiS_5 nanosheet

We calculated the dichroism of Ta_2NiS_5, as shown in **Supplementary Figure 7**. In the visible range, the maximum in-plane $|\Delta k|$ is ~2.0 and the corresponding value for out-of-plane is ~2.7. These values are much larger than other in-plane anisotropic vdW materials, such as GeSe (Δk of 0.90 at ~470 nm)[16], and ZrS_3 (Δk of 0.78 at ~500 nm)[17]. In the MIR range, the maximum in-plane and out-of-plane $|\Delta k|$ values are 0.357 and 0.48 at ~2.5 μm, respectively, which are the highest reported dichroism among anisotropic vdW crystals, to the best of our knowledge.

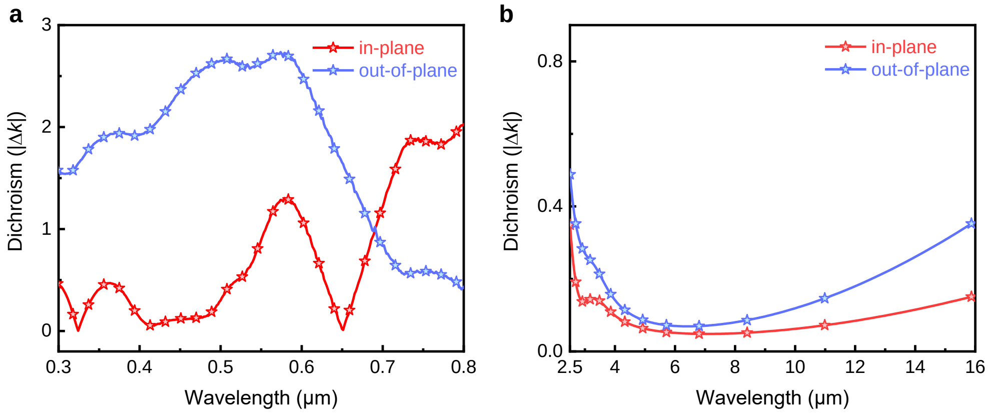

Supplementary Figure 7. The absolute dichroism ($|\Delta k|$) in **a** visible and **b** mid-infrared (MIR) spectral region.

It is notable an uptick in the k (**Figure 1e** in main text) for *a*- and *c*-axes in the wavelength longer than 10 microns occurs, which is possibly induced by the free carrier absorption rather than phonon. Since the absorption peaks of optical phonons are usually much sharper than the electronic absorption bands, this is not like the situation in our observation of the very broadband absorption in the 10-16 μm. Besides, the reported infrared spectra of Ta_2NiS_5 shows a phonon energy range below 50 meV (i.e., > 24.8 micron)[18], which is much longer than our observed range. The free carrier is another possible origin account for this absorption. In our research, Drude-Lorentz model was applied to describe the optical response in the MIR region, and the fitted plasma frequencies from the Drude part are 1155.75 and 1545.28 cm^{-1} for *c*- and *a*-axis, respectively, which are close to the uptick of the MIR region. This gives us a clue to venture a guess that the absorption originates from free carriers.

Supplementary Note 5: Theoretical calculation of the optical properties of Ta_2NiS_5

We performed density functional theory (DFT) calculations to further study the anisotropic optical properties of Ta_2NiS_5. As shown in **Supplementary Figure 8**, the calculated optical properties including dielectric function, refractive and extinction match well with our experimental results. The shift of peaks relative to the experimental results is likely due to the inadequacy of Perdew-Burke-Ernzerhof (PBE) functional in describing the band gap and optical properties of semiconductors.

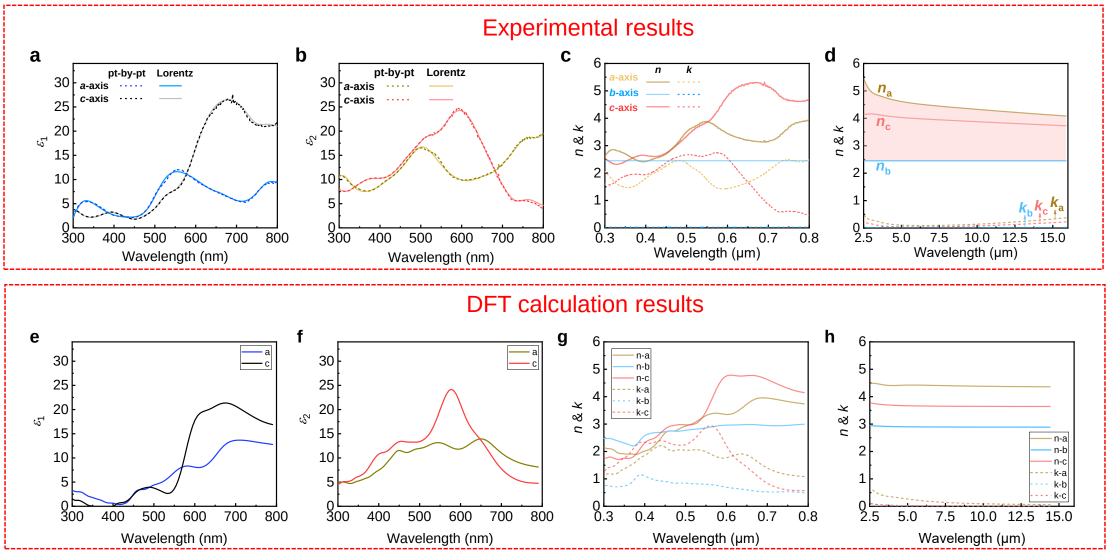

Supplementary Figure 8. Experimental and calculated optical properties of the Ta_2NiS_5. a, b Experimental complex dielectric function along different crystal axis in the visible wavelength. **c, d** Experimental refractive and extinction in the visible and mid-infrared (MIR) wavelength along different crystal axis. **e, f** Density functional theory (DFT) calculated complex dielectric function along different crystal axis in the visible wavelength. **g, h** DFT calculated refractive and extinction in the visible and MIR wavelength along different crystal axis.

Supplementary Note 6: Discussion of fringe formation mechanisms

As discussed in the main text, the real space fringes on the sample surface observed in our s-SNOM measurements are formed by the interference between the photons scattered by the tip (path P_1) and the edge scattering photons (path P_2). In addition to mechanism discussed above and in the main text, **Supplementary Figure 9a** depicted other possible interference mechanisms. In path P_4, the tip-launched waveguide modes could be reflected backward to the tip after reaching the sample edge. The interference between path P_1 and P_4 plays an important role in the formation of surface plasmon polaritons (SPPs) and phonon polariton (PhPs) (*eg.* graphene SPPs and α-MoO_3 PhPs). In the analysis of dielectric waveguides, we ignore the contribution of this interference, because the momentum of optical waveguides is small thus the reflectivity is low. Moreover, additional intrinsic loss during the round-trip propagation (from the tip to the edge and back to the tip) further weakens the reflected waveguide propagating. Therefore, we do not consider this mechanism in the analysis of dielectric waveguide fringes formation. Besides, in addition to the waveguide mode excited by the SNOM tip, the edge of the sample will also launch the waveguide mode under laser irradiation (P_3). Because the size of the focused laser is very small, edge excitation is possible only when tip is very close to the sample edge. Meanwhile, the optical paths of edge excitation-tip scattering and tip excitation-edge scattering are same, so edge-launched modes generate the same fringes as the tip-launched modes. Considering the above factors, we also do not consider the influence of edge excitation in the analysis of interference mechanism.

According to **Supplementary Figure 9b**, based on the geometrical optics theory and momentum conservation relationship between incident light and dielectric waveguide modes, the relationship between near-field experiments observed sample surface wavevector q_{obs} and the genuine wavevector q_{wm} is derived in reference[19]

$$q_{obs} = q_{wm}\cos\gamma + k_0 \sin\beta\cos\alpha \tag{13}$$

where k_0 is the free-space wavevector, α is the angle of the incident laser beam relative to the sample surface, β is the sample edge orientation angle. Here in

Supplementary Figure 9b α = 30°, β = 45°. The γ angle satisfies the momentum conservation along the edge direction

$$\gamma = \sin^{-1}((\frac{k_0}{q_{wm}})\cos\alpha\cos\beta) \tag{14}$$

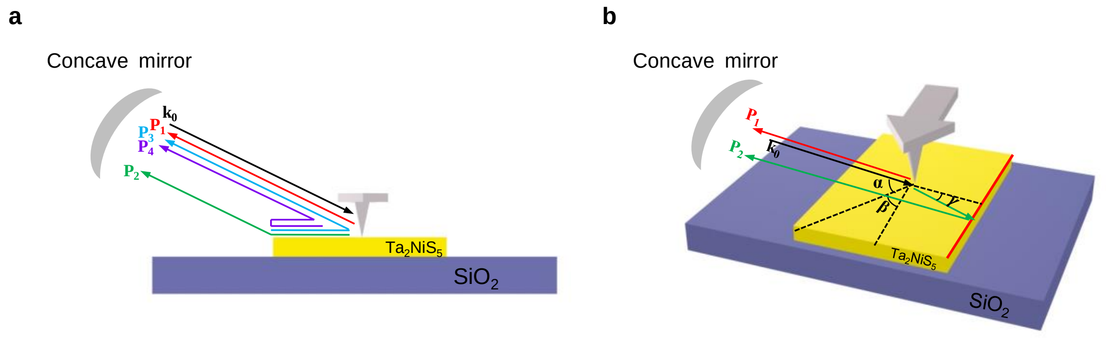

Supplementary Figure 9. Schematic diagrams of fringe formation mechanism. a Illustration of various interference paths. **b** Illustration of the dominant interference path for sample interference fringe formation under β = 45°. The solid red line represents a-axis edge of the sample. The γ angle refers to the waveguide mode propagation angle from tip relative to the sample edge.

Supplementary Note 7: Near-field nanoimaging images

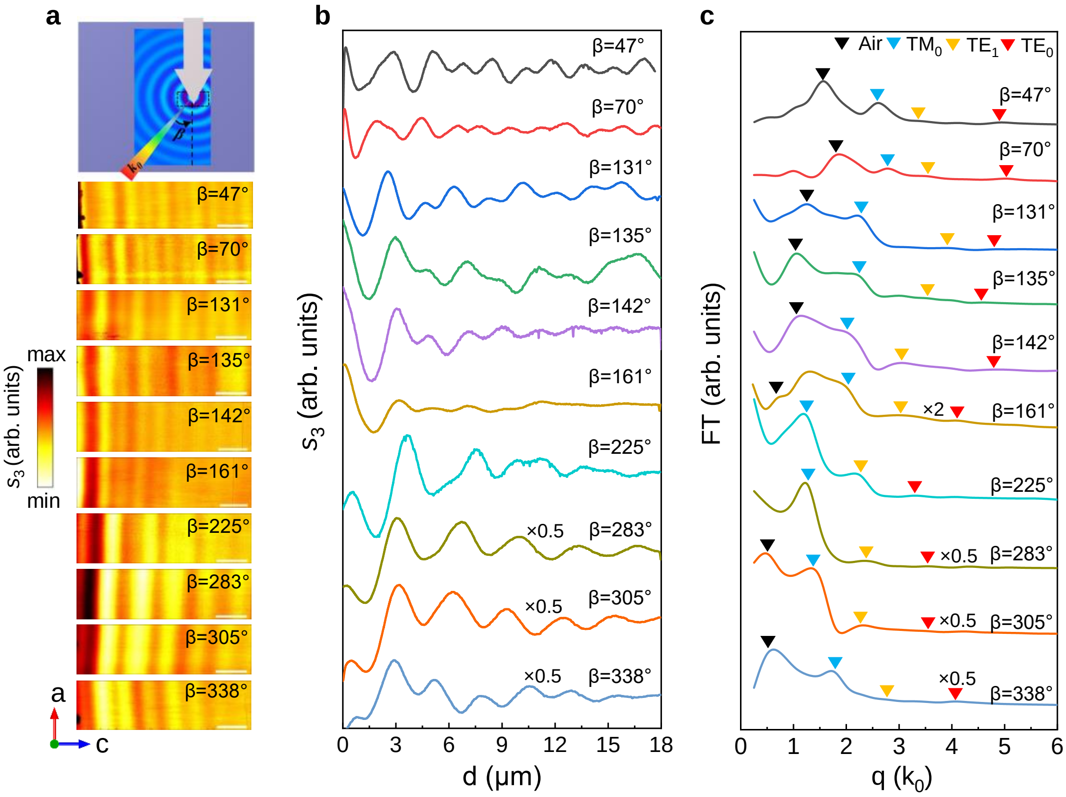

Supplementary Figure 10. Edge-orientation dependance of the waveguide modes under 4.545 µm excitation wavelength in Ta_2NiS_5 flakes. **a** Top-view schematic diagrams of the near-field experimental setup and s-SNOM images of an 881-nm-thick Ta_2NiS_5 flake with excitation wavelength of 4.545 µm under different rotating angles *β*. *β* is the angle between the incident light in-plane projection and the sample's edge (*a*-axis). The scanning region is shown in the black rectangle, closing to the edge of the flake. Scale-bar: 3 µm. **b, c** Real-space fringe profiles and the corresponding Fourier transform (FT) profiles of **a**.

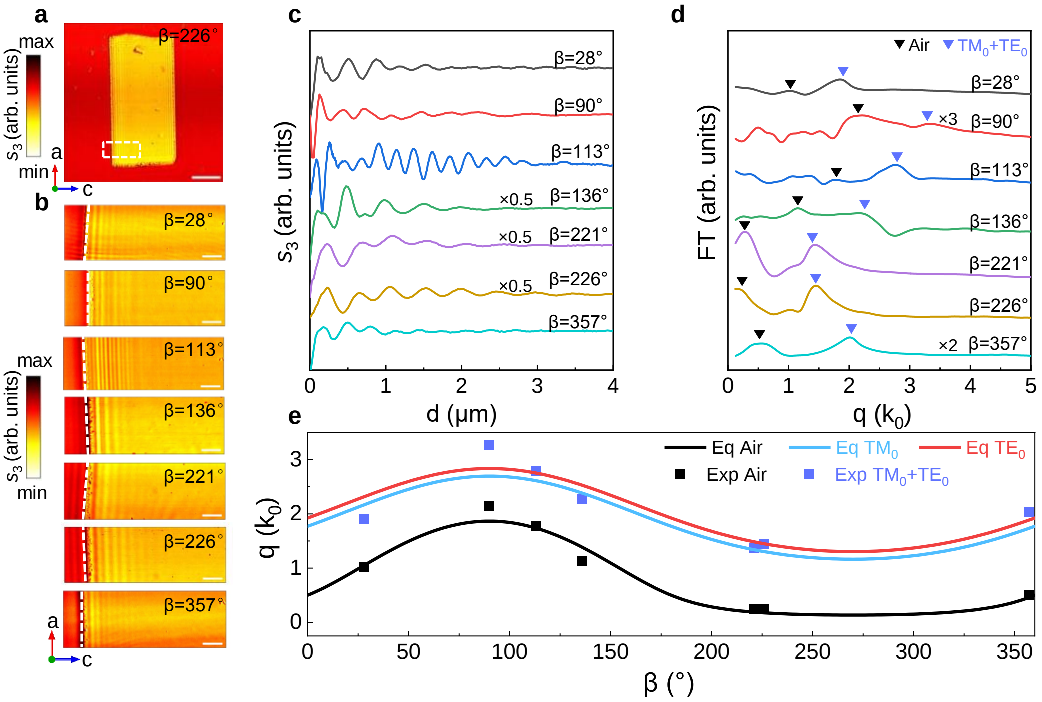

Supplementary Figure 11. Edge-orientation dependent waveguide mode under 633 nm excitation wavelength in Ta_2NiS_5 flake. a Near-field images of an 83-nm-thick Ta_2NiS_5 flake at 633 nm excitation wavelength recorded with β = 226°. Scale-bar: 5 μm. **b** Near-field images with different orientation angles β. β is the angle between the incident light in-plane projection and the sample's edge (*a*-axis).The investigated region is shown in the white rectangle in **a**. The white dashed lines mark the edge of the sample. Scale-bar: 1 μm. **c, d** Real-space fringe profiles and the corresponding Fourier transform (FT) profiles of **b. e** Experimental data points overlaid on the calculated data by theoretical analysis.

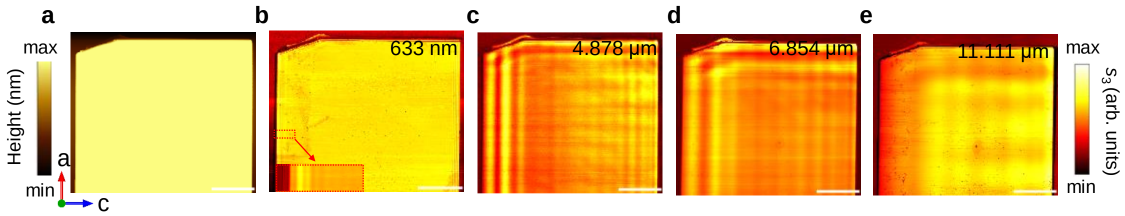

Supplementary Figure 12. Ultra-broadband real-space nanoimaging of Ta_2NiS_5 flake. a AFM topography images of a 638-nm-thick Ta_2NiS_5 flake. **b-e** Near-field amplitude images of Ta_2NiS_5 flake under various excitation wavelengths, labeled in the

upper right corner of each image. scale bar: 10 μm. The left lower panel in **b** shows the propagating waves near the sample edge under visible light illumination.

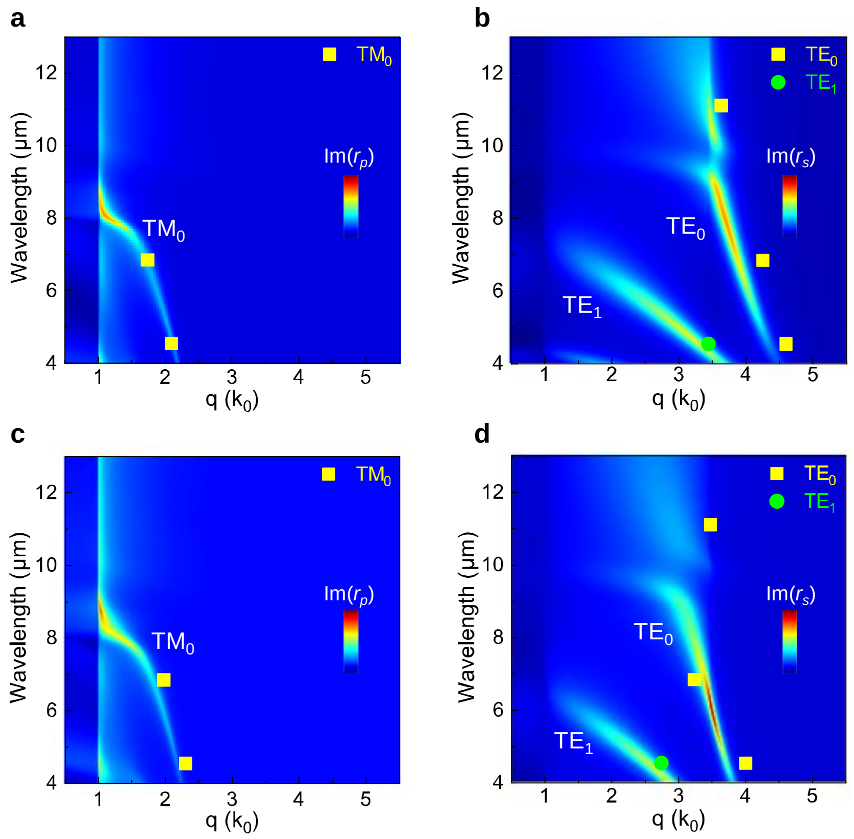

Supplementary Figure 13. Dispersion relationship of 1002-nm-thick Ta_2NiS_5 flake on SiO_2 substrate. a, b Along *c*-axis experimental dispersion data points and theoretical dispersion relations of **a** TM and **b** TE polarized waveguide modes. **c, d** Along *a*-axis experimental dispersion data points and theoretical dispersion relations of **c** TM and **d** TE polarized waveguide modes.

Supplementary Note 8: Extraction of visible in-plane birefringence from near-field imaging

To further confirm the in-plane birefringence of Ta_2NiS_5, we extracted the visible in-plane birefringence based on the near-field results using transcendental equation (1) as mentioned in the main text. The birefringence of the materials is 2.04 under 633 nm excitation wavelength and 0.64 under 785 nm excitation wavelength, which is in good agreement with the spectroscopic ellipsometry results (**Supplementary Figure 14**).

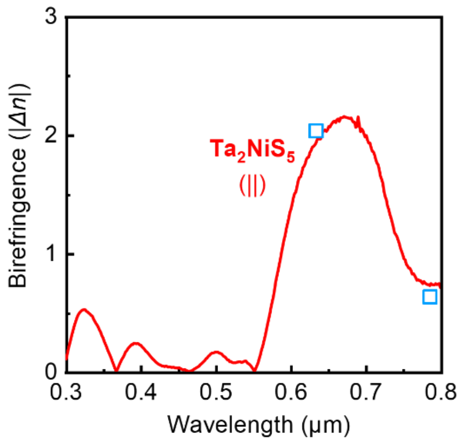

Supplementary Figure 14. The extracted in-plane birefringence data from near-field imaging (blue points) and the spectroscopic ellipsometry (red line) of Ta_2NiS_5.

Supplementary Note 9: Near-field nanoimaging of 1785-nm-thick Ta_2NiS_5 flake at MIR band

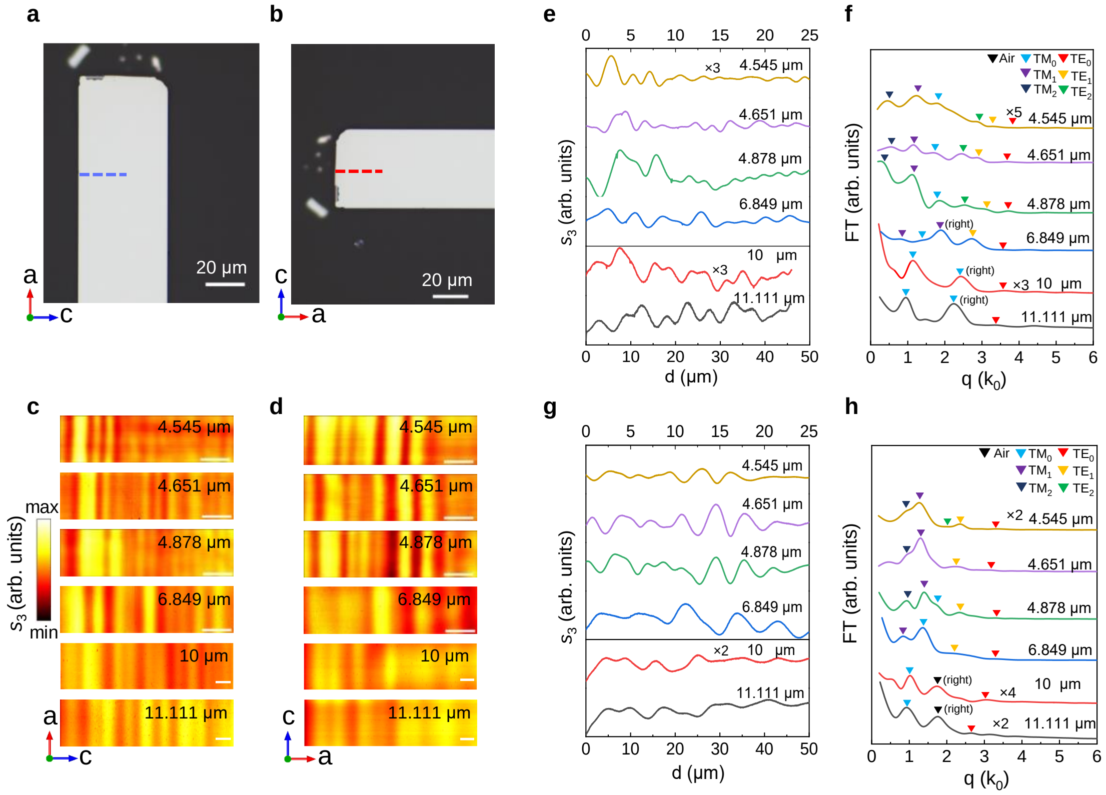

Supplementary Figure 15. Near-field nanoimaging of the in-plane anisotropic waveguide modes at MIR band. a, b Optical microscope images of a 1785-nm-thick Ta_2NiS_5 flake on CaF_2 substrate with **a** *a*-axis on the left **b** *c*-axis on the left. Scale bar: 20 μm. **c, d** Near-field image along *c*-axis (**S15a** blue dash line) and *a*-axis (**S15b** red dash line), respectively. Scale bar: 4 μm. **e, f** Real-space fringe profiles and the corresponding Fourier transform (FT) profiles of **c**. **g, h** Real-space fringe profiles and the corresponding FT profiles of **d.** The label '(right)' in **f** and **h** indicates the FT peak excited by the right edge.

Supplementary Note 10: Extraction of n_b error from near-field imaging

According to the transcendental Equations (2) as shown in the main text, the error of n_b mainly origins from the uncertainty of determining the wave vector q_{wm} from the near-field experimental data. To better support the extracted refractive index, we provided error bars in the near-field experimental dispersion data (**Figure 3 and Figure 4** of the main text), in which the error bars are determined by the half of the full-width at half-maximum (FWHM) of the peak in the Fourier transform (FT) analysis[20]. By solving the transcendental equations, we then obtain the error of the out-of-plane refractive index n_b, as shown in **Supplementary Table 3.** Notably, the extracted refractive index n_b shows a small error within 10%, which indicates that the value of n_b is reliable.

Supplementary Table 3. The extracted n_b value based on the near-field experimental data.

Wavelength	Near-field data point	n_b
4.545 μm	TM_0	2.63±0.08
	TM_1	2.47±0.25
4.651 μm	TM_0	2.56±0.17
	TM_1	2.36±0.13
4.878 μm	TM_0	2.58±0.18
	TM_1	2.45±0.19
6.849 μm	TM_0	2.33±0.16
	TM_1	3.08±0.54
10 μm	TM_0	2.36±0.25
11.111 μm	TM_0	2.40±0.19

Supplementary Note 11: Propagation length of anisotropic waveguide mode in Ta_2NiS_5 flake on CaF_2 substrate

The propagation constant $\beta = \beta_r - i * \beta_i$ of anisotropic waveguide mode in Ta_2NiS_5 was calculated by the mode solver of COMSOL. Thus, the propagation length (L) of anisotropic waveguide mode can be calculated with $L = \frac{1}{2\beta_i}$. **Supplementary Figure 16** shows the calculated propagation length of anisotropic waveguide mode in Ta_2NiS_5 flake on CaF_2 substrate.

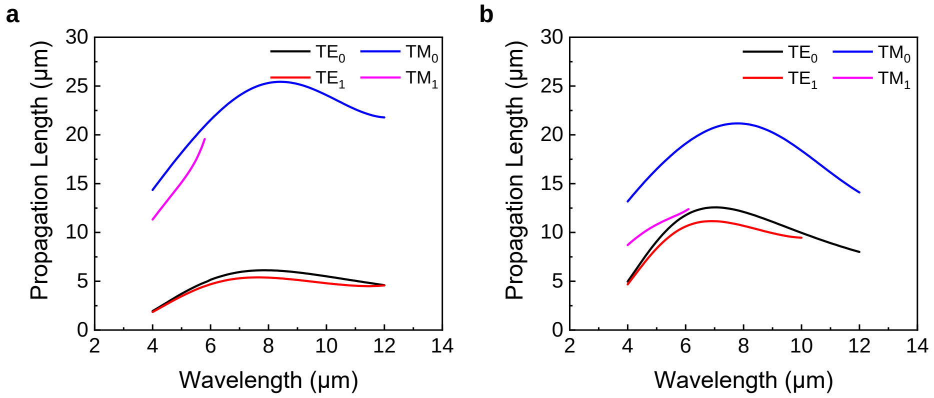

Supplementary Figure 16. Propagation lengths of TE_0, TE_1 and TM_0, TM_1 waveguide modes along *c*-axis (**a**) and *a*-axis (**b**) in a 1785-nm-thick Ta_2NiS_5 sample.

Supplementary Note 12: The thicknesses-dependence of dispersion relationship in Ta_2NiS_5.

Supplementary Figure 17-19 explore the thicknesses-dependent dispersion relationship of the dielectric waveguide modes in Ta_2NiS_5. The TE and TM wavevector and modes number increases as the Ta_2NiS_5 flake thickness increases. **Supplementary Figure 17-18** depict the results of Ta_2NiS_5 flake with various thicknesses on SiO_2 substrate under excitation wavelengths of 633 nm and 4.545 μm, respectively. **Supplementary Figure 19** depicts the results of Ta_2NiS_5 flake with various thicknesses on CaF_2 substrate. We can observe that the phenomena on both substrates are the same.

In the visible regime, the number of waveguide modes increases with the thickness of the Ta_2NiS_5 layer. For a waveguide with a 1002-nm-thick Ta_2NiS_5 sample, there are 6 TE modes and 13 TM modes in theory. Meanwhile, the extracted experimental wave vector in thicker sample falls in the higher order modes[21,22], and the peak of FT analysis may be a mixture of multiple modes. Therefore, it is difficult to identify the corresponding modes by the near-field data for the thick sample. On the contrary, for a thinner sample of 83 nm in thickness, only fundamental TM_0 and TE_0 mode are supported and the experimental data are well consistent with the calculated results.

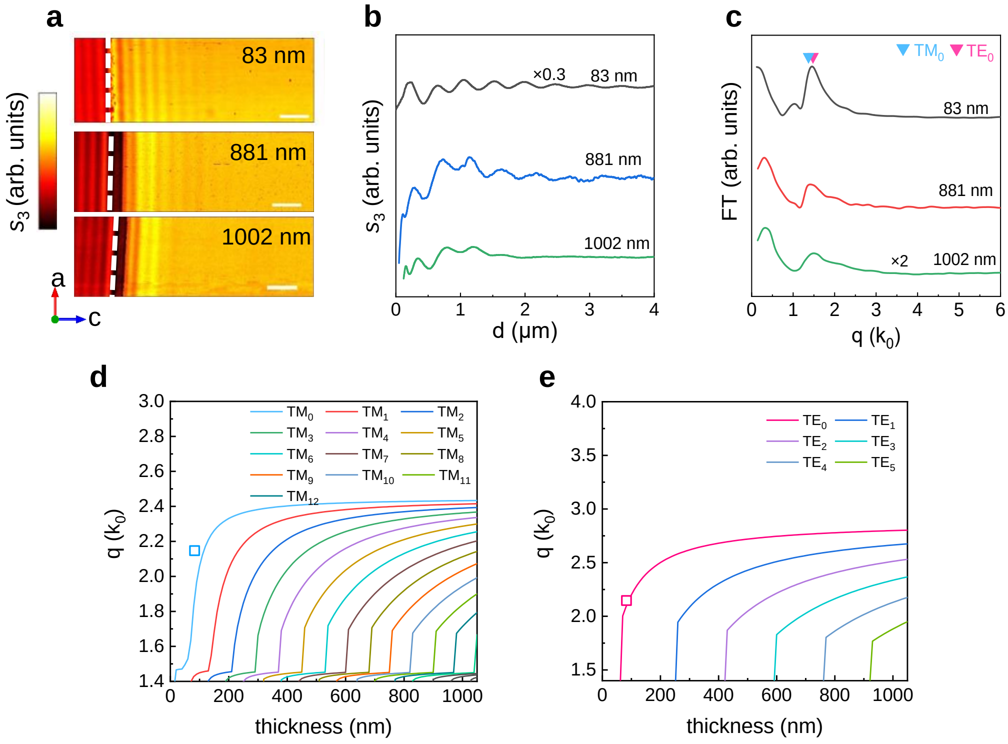

Supplementary Figure 17. Near-field characteristics of Ta_2NiS_5 flakes with various thicknesses on SiO_2 substrate. a Near-field images of Ta_2NiS_5 flake with various thicknesses. The excitation wavelength $\lambda = 633$ nm. The Ta_2NiS_5 flakes are placed *a*-axis on the left and fringes are extracted along *c*-axis. Scale bar: 1 μm. **b, c** Real-space fringe profiles and the corresponding Fourier transform (FT) profiles of **a**. **d, e** Experimental dispersion data points and theoretical dispersion relations of the TM- and TE-polarized waveguide modes of **a**.

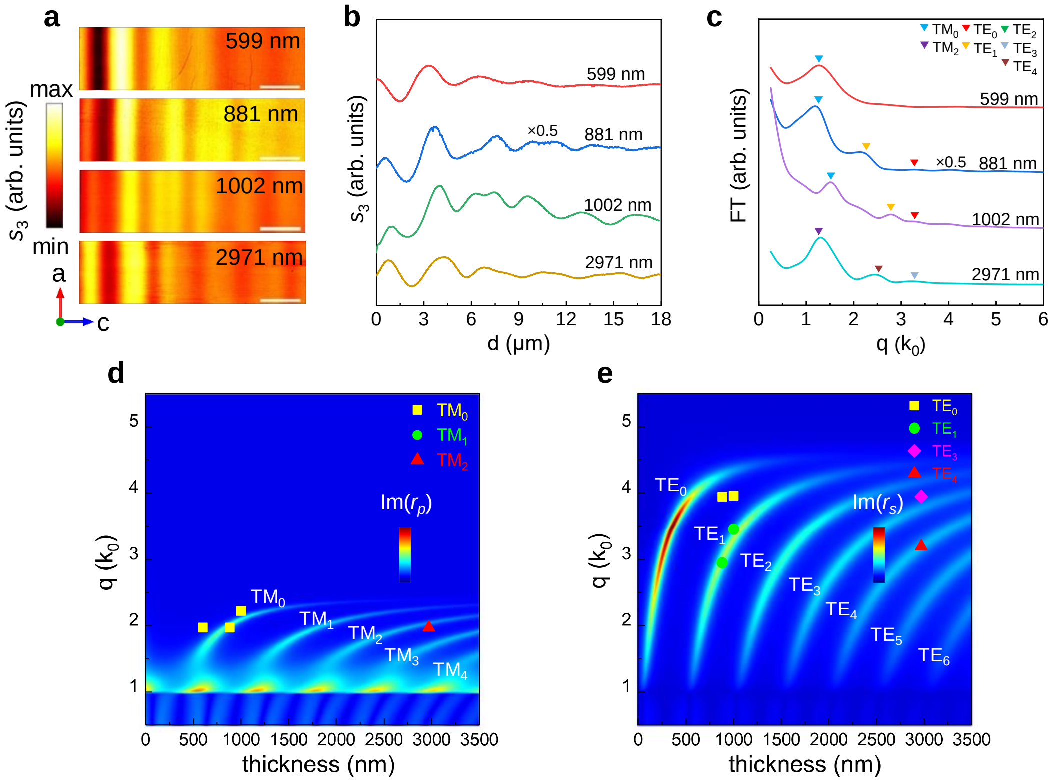

Supplementary Figure 18. Near-field characteristics of Ta_2NiS_5 flakes with various thicknesses on SiO_2 substrate. a Near-field images of Ta_2NiS_5 flake with various thicknesses. The excitation wavelength λ = 4.545 μm. The Ta_2NiS_5 flake are placed with β = 225° and fringes are extracted along *c*-axis. Scale bar: 3 μm. **b, c** Real-space fringe profiles and the corresponding Fourier transform (FT) profiles of **a**. **d, e** Experimental and theoretical dispersion relations of the TM- and TE-polarized waveguide modes of **a**.

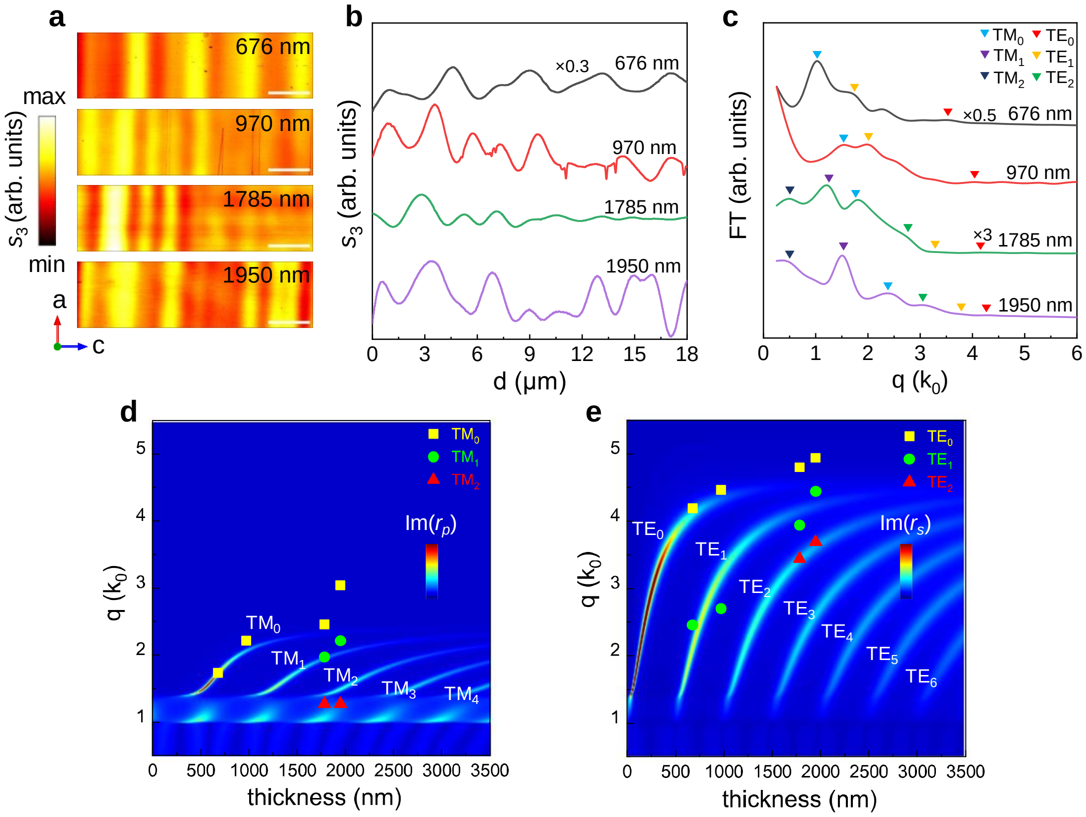

Supplementary Figure 19. Near-field characteristics of Ta_2NiS_5 flakes with various thicknesses on CaF_2 substrate. a Near-field images of Ta_2NiS_5 flake with various thicknesses on CaF_2 substrate. The excitation wavelength λ = 4.545μm. The Ta_2NiS_5 flake are placed with β = 225° and fringes are extracted along *c*-axis. Scale bar: 3 μm. **b c** Real-space fringe profiles and the corresponding Fourier transform (FT) profiles of **a**. **d, e** Experimental and theoretical dispersion relations of the TM- and TE-polarized waveguide modes of **a**.

Supplementary Note 13: Near-field nanoimaging of ternary chalcogenides Ta_2NiSe_5 flake

Supplementary Figure 20 shows the results of real-space nanoimaging of ternary chalcogenides Ta_2NiSe_5 flakes. From **Supplementary Figure 20**, we clearly observe the oscillating fringes that occur inside all the sample flakes within the visible to MIR range. The fringes spacing excited by the left edge is different from that excited by the right edge, indicating that the fringe spacing are related to the angle between sample edge and incident light in-plane projection, which is a typical behavior of dielectric waveguide modes. Thus, ultra-broadband waveguides can be realized by changing the proportion or composition of elements in ternary chalcogenides Ta_2NiSe_5.

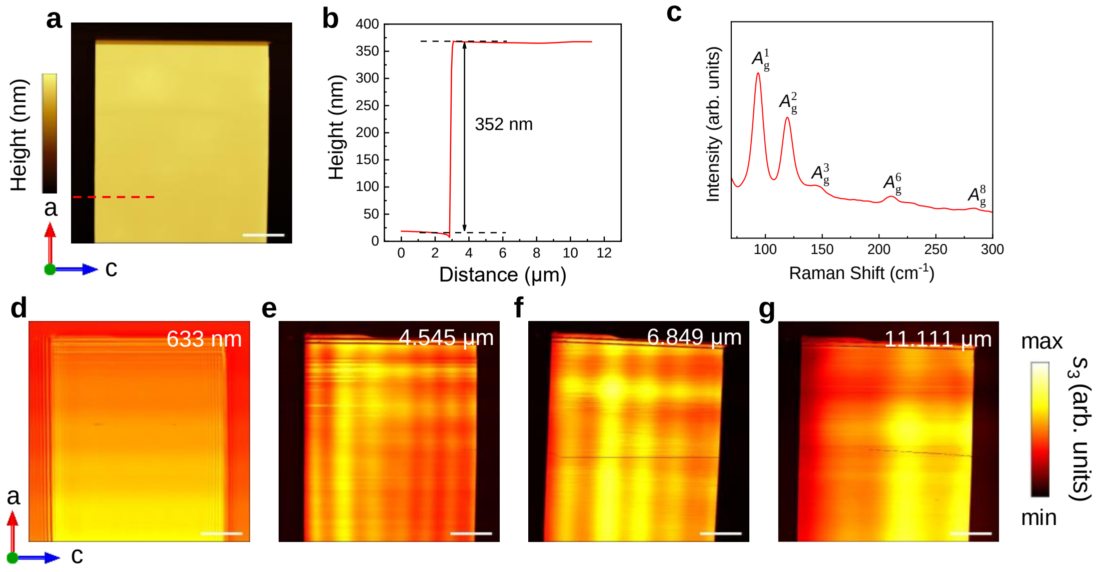

Supplementary Figure 20. Real-space nanoimaging of Ta_2NiSe_5 flake. a AFM topography of the Ta_2NiSe_5 flake. **b** The thickness line profile was extracted along the red dash line in **a**. The sample thickness is measured to be 352 nm. **c** Raman spectra of the Ta_2NiSe_5 flake. **d-g** Near-field amplitude images of Ta_2NiSe_5 flake under various excitation wavelengths, labeled in the upper right corner of each image. Scale bar: 5 μm.

Supplementary Table 4. Comparison of the waveguide modes in different vdW materials.

Material	Wavelength	Category	Propagation Length	Reference
$PtSe_2$	3.97 μm-7.25 μm	Dielectric waveguide	＜ 4 μm	[19]
$SnSe_2$	5.13 μm-6.57 μm	Dielectric waveguide	-	[23]
MoS_2	1530 nm	Dielectric waveguide	-	[22,24]
hBN	632.8 nm	Dielectric waveguide	-	[22]
hBN	1530 nm	Dielectric waveguide	-	[24]
$MoSe_2$	700 nm-919 nm	EPs	~ 12 μm	[25]
SnS	810 nm-961 nm	EPs	＜ 5 μm	[26]
WSe_2	740 nm-900 nm	EPs	~ 3 μm	[27]
WSe_2	701 nm-919 nm	EPs	＜ 2 μm (atomic layer)<br>~ 10 μm (thickness ＞ 30 nm)	[28]
α-MoO_3	9.9 μm-12.2 μm	PhPs	＜ 3.2 μm	[29]
Graphene	11.2 μm	SPPs	＜ 1 μm	[30]
$h^{10}BN$/Silicon hybrid	6.5–7.0 μm<br>1.55 μm	PhPs（6.5–7.0 μm）<br>Dielectric waveguide（1.55 μm）	5.6 μm (under 6.49 μm excition wavelength)	[31]
Ta_2NiS_5	633 nm-11.111μm (Visible—MIR)	Dielectric waveguide	~ 25 μm（MIR）	This work

Supplementary References

1. Sunshine, S. A. et al. Structure and physical properties of the new layered ternary chalcogenides Ta_2NiS_5 and Ta_2NiSe_5. *Inorg. Chem*. **24**, 3611-3614 (1985).
2. Zhuo, X. et al. Dynamical evolution of anisotropic response of type-II Weyl semimetal $TaIrTe_4$ under ultrafast photoexcitation. *Light Sci. Appl.* **10**, 101 (2021).
3. Sun, Y. et al. Low-temperature solution synthesis of few-layer 1T'-$MoTe_2$ nanostructures exhibiting lattice compression. *Angew. Chem. Int. Ed.* **55**, 2830 (2016).
4. Gudelli, V. K. et al. Large bulk photovoltaic effect and second-harmonic generation in few-layer pentagonal semiconductors PdS_2 and $PdSe_2$. *New J. Phys*. **23**, 093028 (2021).
5. Negishi, H. et al. Anisotropic thermal expansion of layered MoO_3 crystals. *Phys. Rev. B* **69**, 064111 (2004).
6. Kawahara, K. et al. Surface structure of novel semimetal WTe_2. *Appl. Phys. Express* **10**, 045702 (2017).
7. Yi, H. et al. The electronic band structure of quasi-one-dimensional van der Waals semiconductors: the effective hole mass of ZrS_3 compared to TiS_3. *J. Phys.: Condens. Matter* **32**, 29LT01 (2020).
8. Ho, C. H. et al. Crystal structure and band-edge transitions of $ReS_{2-x}Se_x$ layered compounds. *J. Phys. Chem. Solids* **60,** 1797–1804 (1999).
9. Lee, K. C. et al. Analog circuit applications based on all-2D ambipolar $ReSe_2$ field-effect transistors. *Adv. Funct. Mater.* **29**, 1809011(2019).
10. Wiedemeier, H. et al. The thermal expansion of GeS and GeTe. *Z. anorg. allg. Chem.* **431**, 299-304 (1977) .
11. Li, L. et al. Strong in-plane anisotropies of optical and electrical response in layered dimetal chalcogenide. *ACS Nano* **11**, 10264-10272 (2017).
12. Su, Y. et al. Highly in-plane anisotropy of thermal transport in suspended ternary chalcogenide Ta_2NiS_5. *Nano Res.* **15**, 6601-6606 (2022).
13. Fujiwara, H. *Spectroscopic Ellipsometry: Principles and Applications*. (John Wiley

& Sons, New York, 2007).

14. Li, W. et al. Broadband optical properties of large-area monolayer CVD molybdenum disulfide. *Phys. Rev. B* **90**, 195434 (2014).
15. Palik, E. D. *Handbook of Optical Constants of Solids*. (Academic Press, Orlando, 1998).
16. Yang, Y. et al. In-plane optical anisotropy of low-symmetry 2D GeSe. *Adv. Opt. Mater.* **7**, 1801311 (2019).
17. Hou, S. et al. Birefringence and dichroism in quasi-1D transition metal trichalcogenides: direct experimental investigation. *Small* **17**, 2100457 (2021).
18. Larkin, T. I. et al. Infrared phonon spectra of quasi-one-dimensional Ta_2NiSe_5 and Ta_2NiS_5. *Phys. Rev. B* **98**, 125113 (2018)
19. Wong, K. P. et al. Edge-orientation dependent nanoimaging of mid-infrared waveguide modes in high-index $PtSe_2$. *Adv. Opt. Mater.* **9**, 2100294 (2021).
20. Babicheva, V. E. et al. Near-field surface waves in few-layer MoS_2. *ACS Photonics* **5**, 2106-2112 (2018).
21. Hermann, R. et al. Mode launching on a multimode slab-waveguide by a plane wave. *Appl. Phys.* **9**, 307-313 (1976).
22. Hu, D. et al. Probing optical anisotropy of nanometer-thin van der waals microcrystals by near-field imaging. *Nat. Commun.* **8**, 1471 (2017).
23. Xue, M. et al. Tin diselenide van der Waals materials as new candidates for mid-infrared waveguide chips. *Nanoscale* **11**, 14113-14117 (2019).
24. Hu, D. et al. Tunable modal birefringence in a low-loss van der Waals waveguide. *Adv. Mater.* **31**, 1807788 (2019).
25. Hu, F. et al. Imaging exciton–polariton transport in $MoSe_2$ waveguides. *Nat. Photon.* **11**, 356-360 (2017).
26. Luan, Y. et al. Imaging anisotropic waveguide exciton polaritons in tin sulfide. *Nano Lett.* **22**, 1497-1503 (2022).
27. Fei, Z. et al. Nano-optical imaging of WSe_2 waveguide modes revealing light-exciton interactions. *Phys. Rev. B* **94**, 081402 (2016).
28. Hu, F. et al. Imaging propagative exciton polaritons in atomically thin WSe_2

waveguides. *Phys. Rev. B* **100**, 121301 (2019).

29. Ma, W. et al. In-plane anisotropic and ultra-low-loss polaritons in a natural van der Waals crystal. *Nature* **562**, 557-562 (2018).
30. Fei, Z. et al. Gate-tuning of graphene plasmons revealed by infrared nano-imaging. *Nature* **487**, 82-85 (2012).
31. He, M. et al. Guided mid-IR and near-IR light within a hybrid hyperbolic-material/silicon waveguide heterostructure. *Adv. Mater*. **33**, 2004305 (2021).
